# Increased Expression of sST2 in Early HIV Infected Patients Attenuated the IL-33 Induced T Cell Responses

**DOI:** 10.3389/fimmu.2018.02850

**Published:** 2018-12-04

**Authors:** Xian Wu, Yao Li, Cheng-Bo Song, Ya-Li Chen, Ya-Jing Fu, Yong-Jun Jiang, Hai-Bo Ding, Hong Shang, Zi-Ning Zhang

**Affiliations:** ^1^NHC Key Laboratory of AIDS Immunology, Department of Laboratory Medicine, The First Affiliated Hospital of China Medical University, Shenyang, China; ^2^Department of Laboratory Medicine, The First Affiliated Hospital of Xiamen University, Xiamen, China; ^3^Clinical and Emergency Medical Laboratory Department, The First Hospital of Shanxi Medical University, Taiyuan, China; ^4^Collaborative Innovation Center for Diagnosis and Treatment of Infectious Diseases, Hangzhou, China; ^5^Key Laboratory of AIDS Immunology of Liaoning Province, The First Affiliated Hospital of China Medical University, Shenyang, China; ^6^Key Laboratory of AIDS Immunology, Chinese Academy of Medical Sciences, Shenyang, China

**Keywords:** IL-33, ST2, T cell response, IFN-γ, HIV infection

## Abstract

T cell responses were less functional and persisted in an exhausted state in chronic HIV infection. Even in early phase of HIV infection, the dysfunction of HIV-specific T cells can be observed in rapid progressors, but the underlying mechanisms are not fully understood. Cytokines play a central role in regulating T cell function. In this study, we sought to elucidate whether IL-33/ST2 axis plays roles in the regulation of T cell function in HIV infection. We found that the level of IL-33 was upregulated in early HIV-infected patients compared with that in healthy controls and has a trend associated with disease progression. *In vitro* study shows that IL-33 promotes the expression of IFN-γ by Gag stimulated CD4+ and CD8+T cells from HIV-infected patients to a certain extent. However, soluble ST2 (sST2), a decoy receptor of IL-33, was also increased in early HIV infected patients, especially in those with progressive infection. We found that anti-ST2 antibodies attenuated the effect of IL-33 to CD4+ and CD8+T cells. Our data indicates that elevated expression of IL-33 in early HIV infection has the potential to enhance the function of T cells, but the upregulated sST2 weakens the activity of IL-33, which may indirectly contribute to the dysfunction of T cells and rapid disease progression. This data broadens the understanding of HIV pathogenesis and provides critical information for HIV intervention.

## Introduction

Powerful and effective HIV-1- specific CD4+ and CD8+ T cell immune responses are generally regarded as the principal antiviral immune activity against HIV (1). However, T cell responses are less functional and persist in an exhausted state in chronic HIV infection. Even in early phase of HIV infection, the dysfunction of HIV-specific T cells was observed in a group of patients (“rapid progressors”) who experienced a sharp decline of CD4+T cells after a short period of infection (2, 3). The underlying mechanisms for the dysfunction of T cells in early HIV infection have not been clearly elucidated.

Cytokines play central roles in regulating T cell function. Previous studies have revealed the role of the cytokine cascade in acute HIV infection and disease progression (4, 5). But the role of some cytokines, which are considered crucial immune modulators with pleiotropic activities, has not been clearly elucidated in early HIV infection (6, 7). IL-33, a recently discovered member of IL-1 family, exercising its role as an alarm signal (alarmin) in response to cellular damage induced by infection or injury to alert immune cells expressing the ST2 receptor (IL-1RL1) (8). The immune-regulation function of IL-33 was initially described in TH2 immune responses and associated diseases (9). Subsequently, studies performed over the past several years have uncovered important roles that IL-33 plays in innate and adaptive immune responses by enhancing natural killer cells, Th1, and CD4 and CD8+T cell functions (8, 10). Bonilla et al. revealed that IL-33 is necessary for potent CD8+ T cell responses to replicating prototypic RNA and DNA viruses in mice (11), suggesting that IL-33 has the potential to boost CD8+ T cell immune responses.

Given the importance of the IL-33/ST2 axis in chronic infection, studies have been conducted to study the relationship that plasma IL-33 and the soluble form of ST2 (sST2) have with HIV disease progression. sST2, which binds free IL-33 without signal transmission, serves as a decoy receptor to locally limit “off target” IL-33 activity, thus avoiding inappropriate inflammatory responses (12). The first study of IL-33 in HIV-infected patients showed that the level of IL-33 was decreased in patients compared with controls (13). Other groups showed that IL-33 was either unchanged (14) or increased (15) in HIV treatment-naive patients compared with controls. Unlike the inconsistent finding of IL-33, most of the studies found that untreated, HIV-infected patients had higher sST2 levels compared with controls (13, 14, 16), although one study showed that the level of sST2 was no obvious difference between patients with HIV and control subjects (17). The expression of sST2 was correlated with, diastolic dysfunction, and all-cause mortality in HIV infection (17, 18). During anti-retroviral therapy, both IL-33 and sST2 levels were decreased (15). In early HIV-infected patients, Mehraj et al. reported an elevated sST2 but not IL-33 level in comparison with controls and found that longer treatment duration initiated during CHI normalized sST2, whereas early antiretroviral therapy had no impact (14). sST2 levels have been revealed to be positively correlated with the percentage of T cells expressing activation and exhaustion markers (14). Therefore, the overall alteration of IL-33 in HIV infection is controversial, and the role of the IL-33/ST2 axis in the regulation of T cell function in HIV infection needs to be further investigated.

In this study, we assessed the plasma IL-33 and sST2 levels in early HIV infection (EHI). We found an elevated IL-33 level in early HIV-infected patients and IL-33 can promote the HIV specific T cell responses measure by IFN-γ secretion. We found the sST2 was also increased in early HIV infected patients, especially in those with progressive infection. *In vitro* study showed that sST2 decreased the augment of T cell function by IL-33.

## Materials and Methods

### Patient Selection

Forty-four treatment-naïve, early HIV-infected patients were enrolled in this study. HIV-1 acquisition within the previous 6 months was defined as EHI. All patients were men who have with sex with men (MSM). Both IL-33 and sST2 levels in the plasma were detected at ~120 days (110 ± 27 days) of HIV infection. Twenty HCs were included in this study. The demographic information and clinical characteristics of the subjects are listed in Table 1. There was no difference between the two groups except CD4+T cells. The ethical review committee from The First Hospital of China Medical University approved the collection of blood samples from HIV-infected patients and healthy controls. Informed consent for participation in the study was obtained from all patients.

**Table 1:** Demographic and clinical characteristics of subjects.

**Characteristic**	**HIV infected patients**	**Healthy controls**
Subject no.	44	20
Age (years, Mean ± SD)	27 ± 7	26 ± 4
Male (No,%)	44 (100%)	20 (100%)
Han Ethnic (No,%)	42 (95%)	20 (100%)
CD4 (cells/μL, Mean ± SD)	491 ± 217	747 ± 198
CD8 (cells/μL, Mean ± SD)	1266 ± 683	N/A
VL (Log copies/ml, Mean ± SD)	4.15 ± 1.03	N/A
Estimated date of infection (days, Mean ± SD)	110 ± 27	N/A

### Detection of IL-33 and ST2 Level in Plasma

Plasma was aliquoted into cryogenic vials and stored at −80°C. IL-33 and sST2 levels were measured using the ELISA Kit (R&D Systems) according to manufacturer's instructions.

### Cell Culture and IL-33 Treatment

Peripheral blood mononuclear cells (PBMCs) were isolated from whole blood by Ficoll centrifugation. CD4^+^ and CD8^+^ T cells were purified using negative selection beads (Stem Cell Technologies). The sorted CD4+T and CD8+T cells were seeded with a 96-well U plate and were stimulated with Gag pool (2 μg/mL, Sigma) or CEF (Cytomegalovirus, Epstein-Barr virus, and Influenza A virus) peptide pools (0.03 μg/mL, Miltenyi Biotec). Recombinant IL-33 (R&D Systems) was added to the culture at concentrations of 0, 0.1, 1, 10, and 100 ng/mL (19, 20), respectively for 3 days at 37°C. Cultured cells were kept in a 5% CO2 incubator. Golgistop (500 μg/mL, BD Biosciences) was added during the final 5 h.

### Anti-ST2 Antibody Treatment

For CD3+T cell purification, PBMCs were stained with PerCP-Cy5.5 conjugated anti-CD3 and were sorted with a FACS Aria flow cytometer (BD Biosciences). Recombinant IL-33 (1 ng/mL) was added to the sorted CD3+T cells and cultured for 3 days. In the meantime, Anti-ST2 antibody (R&D Systems) were added to the culture at concentrations of 0.2, 2, and 20 μg/mL. Goat IgG (20 μg/mL, R&D Systems) were added as a control. Cells were cultured for 3 days and Golgistop (500 μg/mL, BD Biosciences) was added during the final 5 h.

### Flow Cytometry

Flow cytometry was used to detect the effect of IL-33 and anti-ST2 antibody treatment on IFN-γ expression in T cells. For IL-33 treatment, isolated CD4+ or CD8+T cells were intracellularly stained with APC-conjugated anti-IFN-γ (BD Biosciences) and LIVE/DEAD Violet (Invitrogen, Carlsbad, CA) at the end of culture. For ST2 treatment, isolated CD3+T cells were stained with PE-conjugated anti-CD8 and intracellularly stained with APC-conjugated anti-IFN-γ and LIVE/DEAD Violet at the end of culture. Cells were acquired on an LSRII flow cytometer and analyzed by FlowJo 7.6 software.

### IFN-γ ELISPOT Assay

CD4+ T cells were depleted from PBMCs by using anti-CD4 MAb-coated magnetic beads (Biolegend, San Diego, California, USA) as described in the manufacturer's instructions. The Human IFN-γ ELISpot Kit (Mabtech) was used to detect secretion of IFN-γ according to the instruction manual. 2 × 10^5^ CD4+T cell depleted PBMCs were added per well in duplicate in a volume of 200 μl, and the HIV-1 gag peptide pools or CEF peptide pools (20 μg/ml, ProImmune) were added for 20 h. Anti-CD3/CD28 beads (3 μg/ml) was used as a positive control, and negative controls consisted of cells without stimuli. Analysis was done using the ImmunoSpot plate reader.

### Statistical Analysis

The SPSS 17.0 software package was used for statistical analysis. The Mann–Whitney test was used to determine the differences between HIV patients and HCs. Correlations between variables were evaluated using the Spearman rank correlation test. To compare the change in IFN-γ expression induced by IL-33 or ST2 by intracellular staining, the media-alone condition was compared with that of the IL-33 and/or ST2-treated conditions using a paired *t*-test. To compare IFN-γ secretion between gag peptides stimulated T cells and controls by ELISPOT assay, the numbers of spot forming cells were log transformed and then compared by paired *t*-test. *P-*values under 0.05 were considered statistically significant.

## Results

### Elevated IL-33 Levels in Primary HIV-Infected Patients

**Figure 1:**
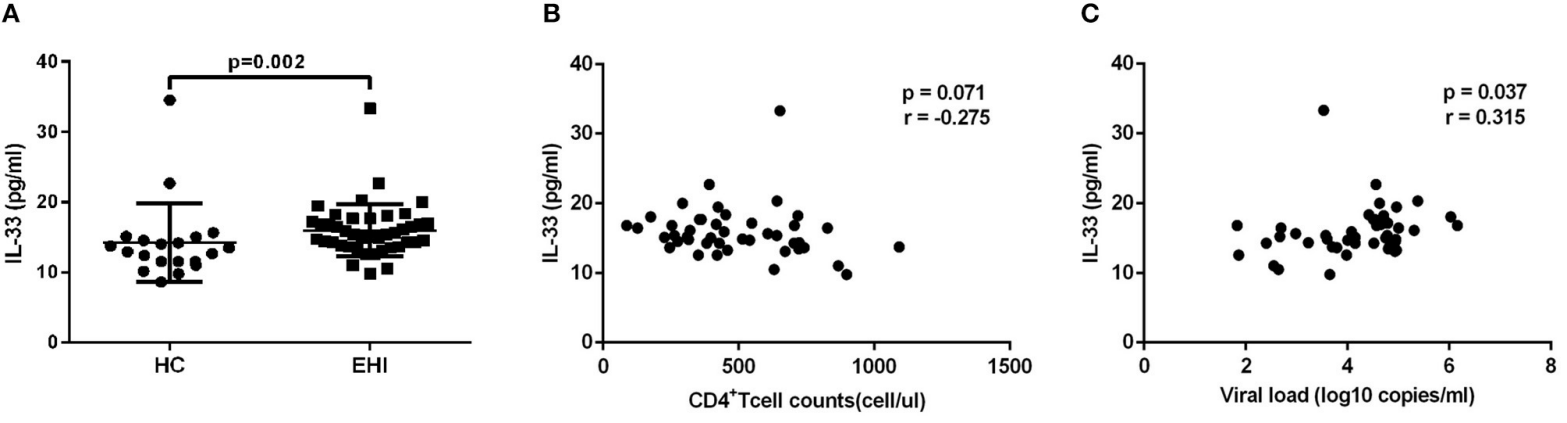
The increased IL-33 level was associated with progression of HIV infection. **(A)** Comparison of the plasma IL-33 level in early HIV infected patients (EHI, 15.96 ± 3.70 pg/mL, *n* = 44) and healthy controls (HC, 14.29 ± 5.60 pg/mL, *n* = 20) using the non-parametric Mann-Whitney test. The relationship between plasma IL-33 and CD4+ T cell counts **(B)**, viral load **(C)** in EHI patients; Spearman's rank correlation coefficients r and *p*-values are indicated.

Firstly, we investigated the plasma IL-33 level in EHI patients enrolled in our study. All these patients had considerably greater IL-33 plasma concentrations (15.96 ± 3.70 pg/mL, *n* = 44) than HCs (14.29 ± 5.60 pg/mL, *n* = 20) using the non-parametric Mann-Whitney test (*P* = 0.002; Figure 1A). We then studied the association of IL-33 levels with disease progression. We found that the expression of IL-33 has a trend of negative correlation with CD4^+^ T-cell counts (*r* = −0.275, *P* = 0.071; Figure 1B) and a trend associated with viral load (*r* = 0.315, *P* = 0.037; Figure 1C).

### IL-33 Promotes the Expression of IFN-γ by Gag and CEF Stimulated CD8+ T Cells From HIV-Infected Patients

**Figure 2:**
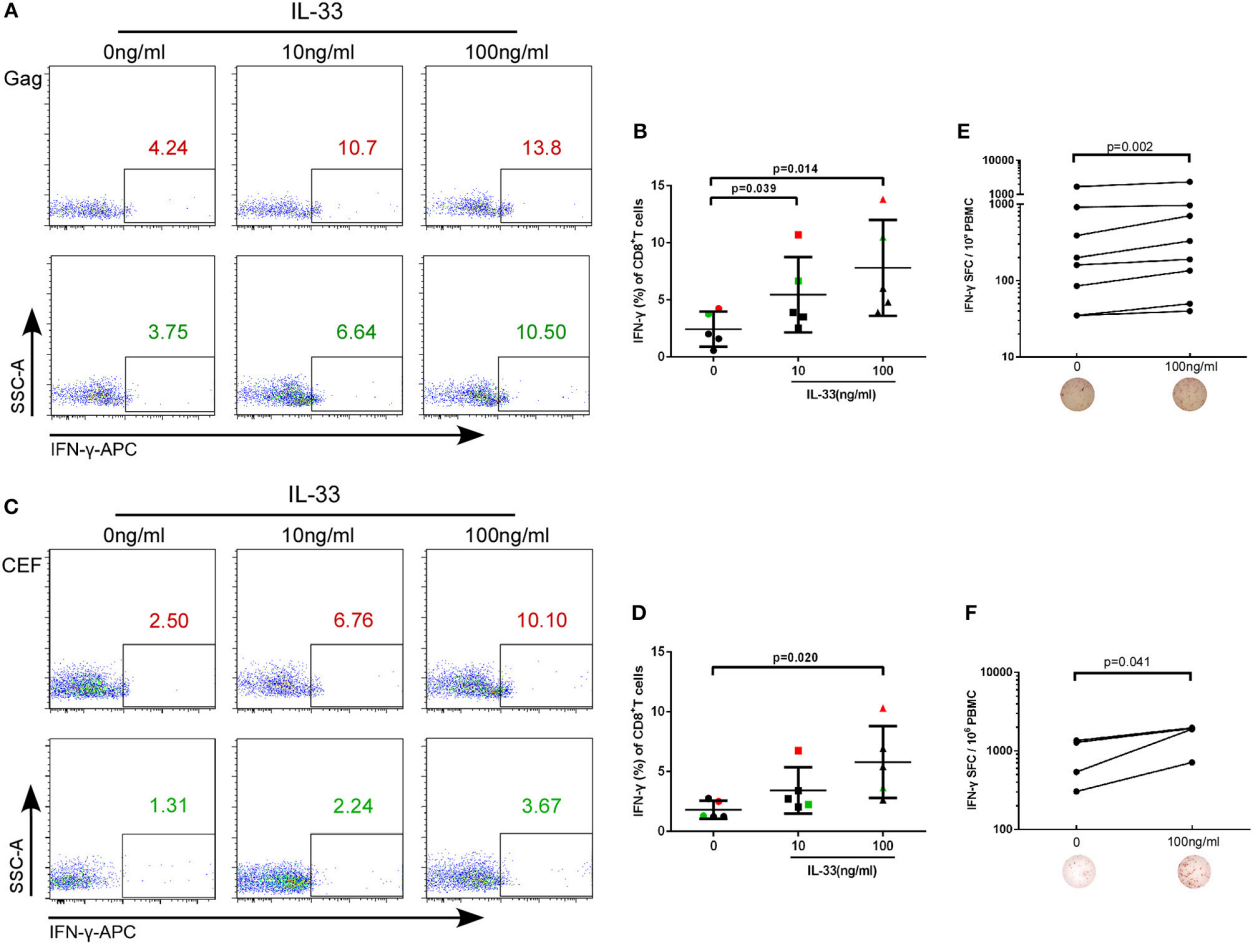
IL-33 increases the expression of IFN-γ by Gag and CEF stimulated CD8^+^ T cells. CD8^+^ T cells were isolated from HIV-1 individuals and treated with Gag peptide pools with rhIL-33 (10 ng/mL and 100 ng/mL) or without IL-33 (0 ng/mL). Intracellular IFN-γ expression was detected by flow cytometer and compared by paired *t*-test (0 ng/mL: 2.44 ± 1.53%; 10 ng/mL: 5.46 ± 3.30%; 100 ng/mL: 7.81 ± 4.20%). Representative flow cytometry dot plot **(A)** and summary data **(B)** were shown. CD8^+^ T cells were isolated from HIV-1 individuals and treated with CEF peptide pools with rhIL-33 (10 ng/mL and 100 ng/mL) or without IL-33 (0 ng/mL). Intracellular IFN-γ expression was detected by flow cytometer and compared by paired *t*-test (0 ng/mL: 1.81 ± 0.75%; 10 ng/mL: 3.44 ± 1.93%; 100 ng/mL: 5.80 ± 3.00%). Representative flow cytometry dot plot **(C)** and summary data **(D)** were shown. CD8^+^ T cells were stimulated with Gag peptide pools **(E)** or CEF peptides **(F)** and IFN-γ secretion was detected by ELISPOT assay. The numbers of spot forming cells (SFC) were log transformed and then compared by paired *t*-test. The number of SFC treated by 100 ng/mL IL-33 were compared with cells without IL-33 stimulation (0 ng/mL).

The rate at which HIV-1 infected individuals progress to AIDS is highly variable and impacted by T cell immunity (21). IL-33 has recently been found to be necessary for enhancing protective antiviral CD8+ T cell responses in mice (11). However, the effect of IL-33 on CD8+T cells in HIV infection has not yet been elucidated. CD4+ and CD8+ T cells from HIV infected patients were isolated and treated with recombinant human IL-33 at different concentrations and stimulated with the Gag peptide pool. Intracellular IFN-γ expression in CD8+T cells was detected at 3 days of culture and was compared by paired *t*-test. We found that compared with cells cultured without IL-33 (2.44 ± 1.53%), Gag-stimulated CD8+T cells secrete more IFN-γ in HIV-infected patients treated with 10 ng/mL (5.46 ± 3.30%, *P* = 0.039) and 100 ng/mL IL-33 (7.81 ± 4.20%, *P* = 0.014, Figures 2A,B). After we confirmed that IL-33 increased the function of HIV-specific CD8+T cells, we sought to know whether IL-33 could also promote the function of CD8+T cells under HIV non-specific stimulant. CEF peptides were added with different concentrations of recombinant IL-33 and the results showed that IFN-γ expression by CD8+T cells was also increased in HIV-infected patients compared with the controls (0 ng/mL, 1.81 ± 0.75%; 100 ng/mL 5.80 ± 3.00%) (*P* = 0.020; Figures 2C,D). To further confirm the function of IL-33 on CD8+T cells, IFN-γ ELISPOT assay was performed. The numbers of spot forming cells were log transformed and then compared by paired *t*-test. We found that IL-33 can increase the IFN-γ secretion by gag (*P* = 0.002, Figure 2E) and CEF peptide pools (*P* = 0.041, Figure 2F) stimulated CD8+T cells. Although IL-33 can augment the function of CD8+T cells in HIV infection, we found that IL-33 cannot lead to a strong increase of T cell function. According to our results, IL-33 can promote the immune response of CD8+T cells induced by both HIV-specific and non-specific stimulation as measured by IFN-γ expression.

### IL-33 Promotes the IFN-γ Expression by Gag and CEF Stimulated CD4+ T Cells From HIV-Infected Patients

**Figure 3:**
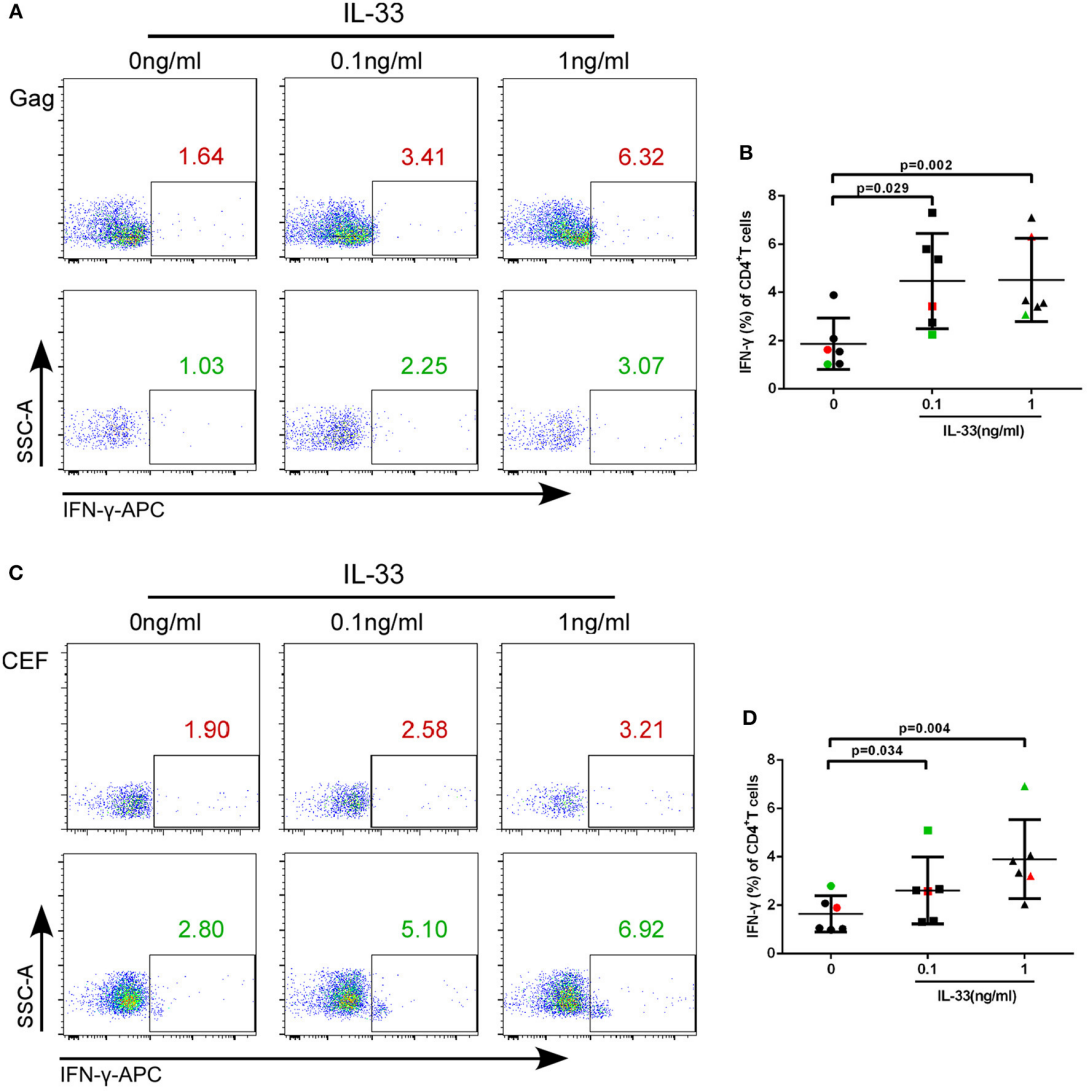
IL-33 increases the secretion of IFN-γ by Gag and CEF stimulated CD4^+^ T cells. CD4+ T cells were isolated from HIV-1 individuals and treated with Gag peptide pools with rhIL-33 (0.1 ng/mL and 1 ng/mL) or without IL-33 (0 ng/mL). Intracellular IFN-γ expression was detected by flow cytometer and compared by paired *t*-test (0 ng/mL: 1.88 ± 1.06%; 0.1 ng/mL: 4.48 ± 1.98%; 1 ng/mL: 4.52 ± 1.73%). Representative flow cytometry dot plot **(A)** and summary data **(B)** were shown. CD4+ T cells were isolated from HIV-1 individuals and treated with CEF peptide pools with rhIL-33 (0.1 ng/mL and 1 ng/mL) or without IL-33 (0 ng/mL). Intracellular IFN-γ expression was detected by flow cytometer and compared by paired *t*-test (0 ng/mL: 1.64 ± 0.74%; 0.1 ng/mL: 2.60 ± 1.38%; 1 ng/mL: 4.06 ± 1.60%). Representative flow cytometry dot plot **(C)** and summary data **(D)** were shown.

We further studied whether IL-33 could affect the function of CD4+T cells in HIV-infected patients. We added varying concentrations of recombinant human IL-33 under the presence of Gag pool peptides to observe the changes in IFN-γ expression from isolated CD4+T cells by paired *t*-test. The results showed that in comparison with cells cultured without IL-33 (1.88 ± 1.06%), Gag peptide-treated CD4+ T cells secrete more IFN-γ in HIV-infected patients treated with 0.1 ng/mL (4.48 ± 1.98%, *P* = 0.029) and 1 ng/mL IL-33 (4.52 ± 1.73%, *P* = 0.002; Figures 3A,B).

We then sought to know whether IL-33 could also promote the function of CD4+T cells under CEF stimulation. The results showed that the production of IFN-γ by CD4+T cells was increased in the presence of 0.1 ng/mL (2.60 ± 1.38%, *P* = 0.034) and 1 ng/mL IL-33 (4.06 ± 1.60%, *P* = 0.004) compared with the controls (1.64 ± 0.74%) (Figures 3C,D).The results suggested that IL-33 can increase the function of CD4+T cells induced by both HIV antigen-specific and non-specific stimulation as measured by IFN-γ expression.

### Expression of sST2 in Plasma of Patients With EHI

**Figure 4:**
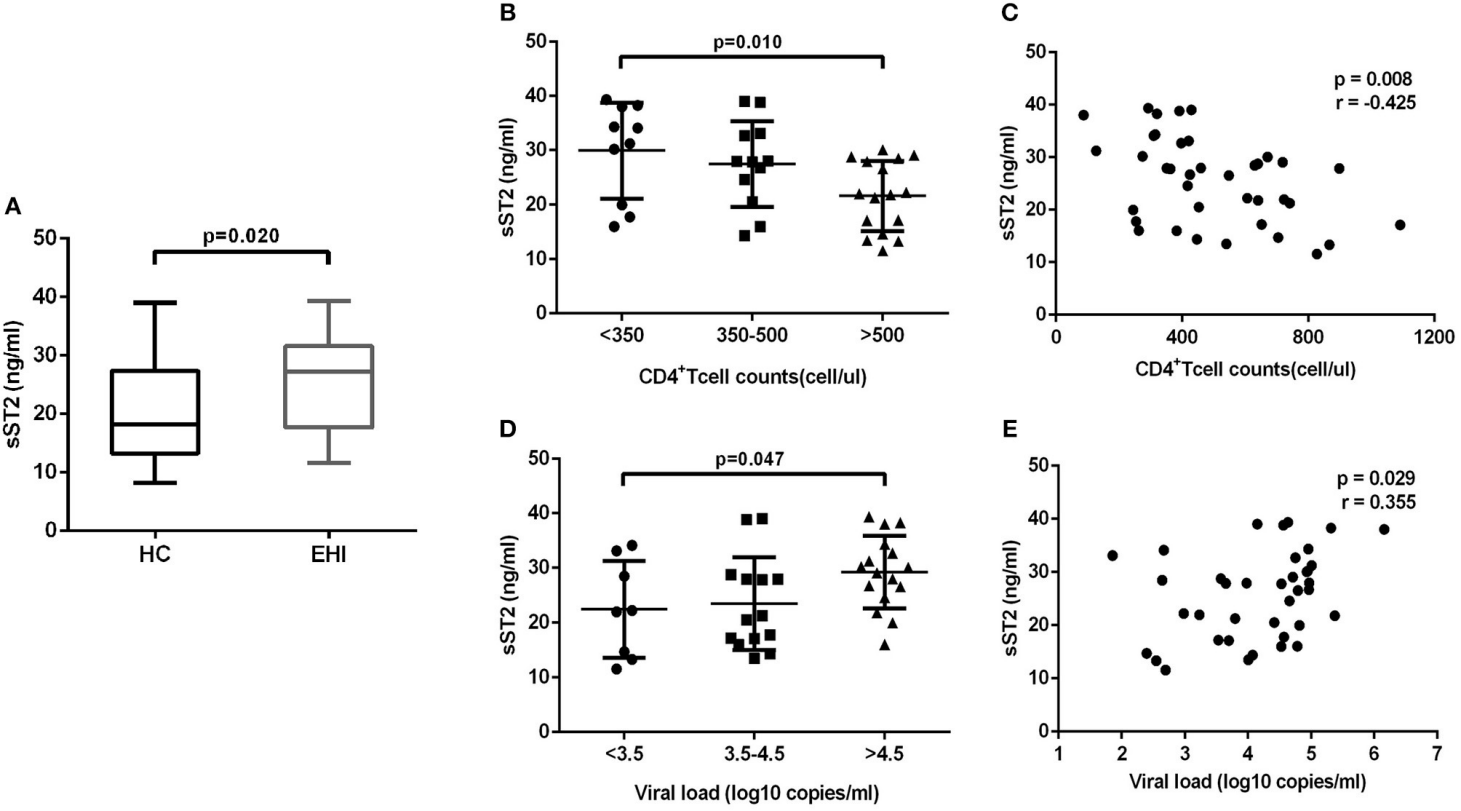
EHI patients demonstrate high serum sST2 levels. **(A)** Comparison of the plasma sST2 level in EHIs (*n* = 38) and HCs (*n* = 20), by Mann-Whitney test. **(B)** Comparison of plasma sST2 in EHIs in CD4+ T cell count groups (<350; 350–500; >500 cells/μL); **(D)** Comparison of plasma IL-33 in EHIs in different viral load groups (<3.5; 3.5–4.5; >4.5 log copies/mL), by Mann-Whitney test. The relationship between plasma IL-33 and CD4+ T cell counts **(C)**, viral load **(E)** in EHI patients; Spearman's rank correlation coefficients r and *p*-values are indicated in figures.

The above-mentioned results revealed that IL-33 could promote the HIV-specific T cell function, which is the dominant component of immunization to control the viral infections (14). But we have found that the increase of IL-33 secretion during EHI was not correlated with higher CD4+T cells. In contrast, we found that IL-33 level was significantly correlated with viral load. We questioned why the increased IL-33 in HIV-infected patients did not protect them from disease progression, as IL-33 mediates effective T cell responses. As the member of the interleukin-1 receptor family, sST2 acts as a decoy receptor for IL-33, which can inhibit the function of IL-33. We postulated that the function of IL-33 on T cells was masked by sST2 in HIV infection and examined the sST2 level in plasma of the HIV infected patients in EHI. We found that the secretion of sST2 was markedly enhanced in EHI patients (25.64 ± 8.19 ng/mL, *n* = 38) compared with HCs (20.21 ± 8.41 ng/mL, *n* = 20) (*P* = 0.020, Figure 4A) using the non-parametric Mann-Whitney test. Although patients enrolled in our study were in the early stage of HIV infection (110 ± 27 days post infection), they had quite different levels of CD4+T cells and viral loads when plasma sST2 was detected. We then studied the association of levels of sST2 with disease progression. We divided patients into three groups according to CD4+ T cell counts and viral loads. The results showed that sST2 was significantly higher in patients with seriously reduced CD4+ T cell group (*P* = 0.010; Figure 4B) and high viral load group (*P* = 0.047; Figure 4D). sST2 was negatively correlated with CD4+ T cell counts (*r* = −0.425, *P* = 0.008, Figure 4C) and positively correlated with viral load (*r* = 0.355, *P* = 0.029; Figure 4E). The correlation between elevated sST2 and disease progression in early HIV-infected patients suggested that sST2 may be involved in the regulation of T cell responses by influencing IL-33 activity in HIV infection.

### Anti-ST2 Antibody Attenuated the Effect of IL-33 on T Cells

**Figure 5:**
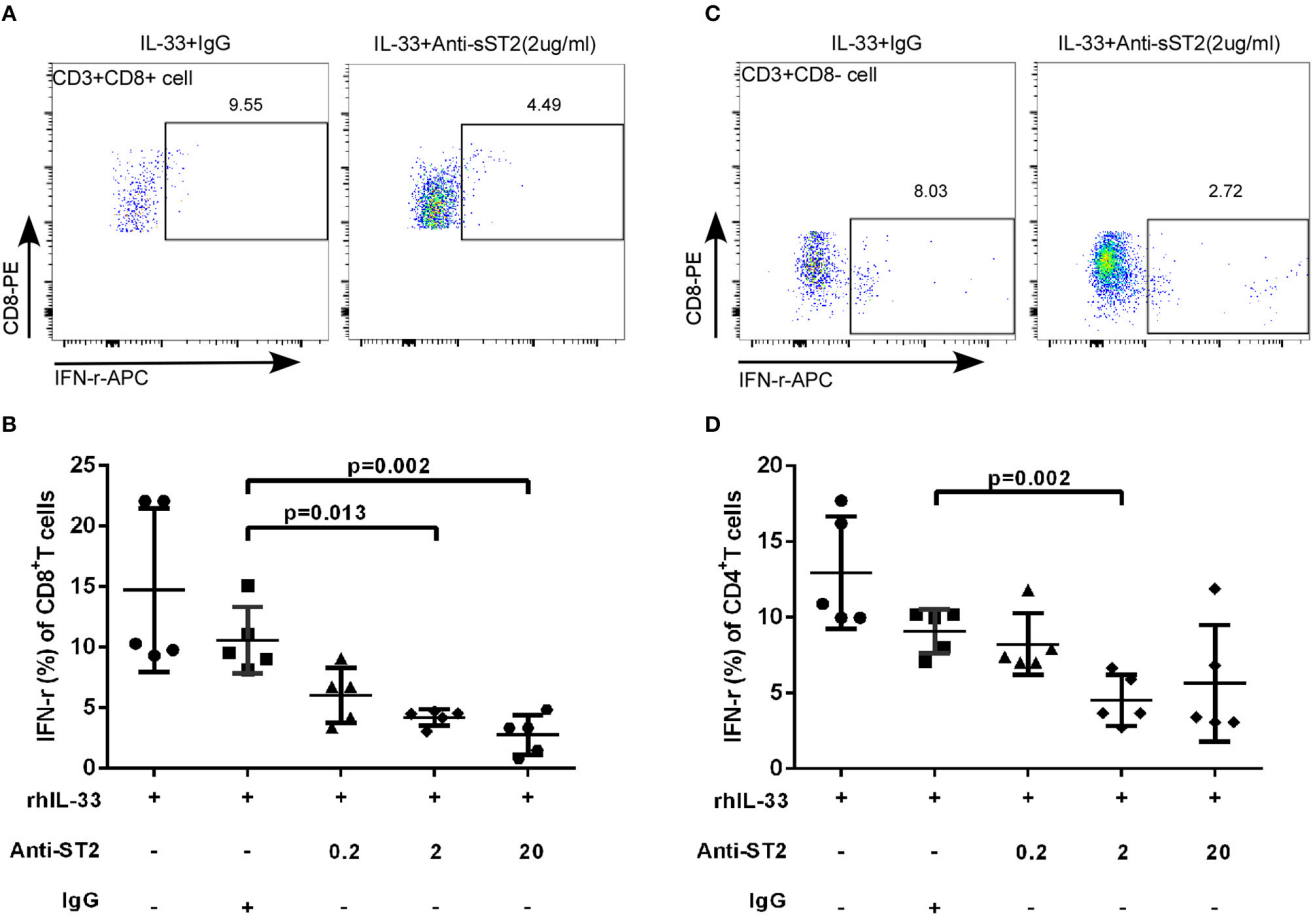
Blocking IL-33 signaling pathway weaken the effect of IL-33 to CD8^+^ and CD4^+^ T cells. CD3+T cells from HIV-infected patients were sorted were treated with rhIL-33 (1 ng/mL) and anti-ST2 with the different concentration and cultured for 3 days and Golgistop (500 μg/mL) was added during the final 5 h. Intracellular IFN-γ expression by CD8+T cells was detected by flow cytometer and compared by paired *t*-test (Goat IgG: 10.58 ± 2.75%, 0.2 μg/mL: 6.02 ± 2.28%, 2 μg/mL: 4.19 ± 0.67%, 20 μg/mL: 2.76 ± 1.63%). Representative flow cytometry dot plot **(A)** and summary data **(B)** were shown. Intracellular IFN-γ expression by CD4+ (CD8-T cells) was detected by flow cytometer and compared by paired *t*-test (Goat IgG: 9.09 ± 1.44%, 0.2 μg/mL: 8.23 ± 2.03%, 2 μg/mL: 4.52 ± 1.67%, 20 μg/mL: 5.65 ± 3.84%). Representative flow cytometry dot plot **(C)** and summary data **(D)** were shown.

IL-33 activates the signaling pathway by binding to transmembrane receptors ST2 and Interleukin-1 Receptor Accessory Protein (IL1RAP) to form heterodimers (22). To explore whether the increased expression of sST2 attenuates the regulation of T cell function in patients with accelerated disease progression, anti-ST2 antibody was added in the experimental system, which binds to the transmembrane receptor ST2 to block the IL-33 signaling pathway, mimicking the binding of sST2 to IL-33 to block the function of IL-33. We isolated CD3+T cells from HIV-infected patients and treated the cells with Gag pools, IL-33 and different concentrations of anti-ST2. Cells were programmed anti-ST2 with concentrations of 0, 0.2, 2, 20 μg/mL and Goat IgG (negative control) in the presence or absence of IL-33. Compared with Goat IgG control (10.58 ± 2.75%), anti-ST2 antibody can inhibit the IFN-γ secretion by Gag specific CD8+T cells at the concentrations of 2 and 20 μg/mL in the presence of IL-33 by paired *t*-test (2 μg/mL: 4.19 ± 0.67%, *P* = 0.013; 20 μg/mL: 2.76 ± 1.63%, *P* = 0.002; Figures 5A,B). We subsequently measured the IFN-γ levels of CD4+T (CD3+CD8- T) cells. The secretion of IFN-γ in CD4+T cells was significantly reduced by anti-ST2 antibody at the concentration of 2 μg/mL *(*4.52 ± 1.67%) compared with Goat IgG control (9.09 ± 1.44%, *P* = 0.002; Figures 5C,D) by paired *t*-test. Taken together, these results demonstrate that the IL-33-mediated increase of IFN-γ secretion is attenuated by the increased expression of sST2, potentially accounting for the differential effect of IL-33 and sST2, as well as the similar trend of increase in EHI.

## Discussion

It has been well established that T cell immunity plays a central role in controlling HIV and has a pronounced influence on disease progression. In patients with advanced disease progression, dysfunction of T cells showed up early in HIV infection. In this study, we demonstrated that IL-33 enhances HIV-specific CD4+ and CD8+T cell immune responses. However, the elevation of sST2 levels in early HIV-infected patients with progressive disease attenuates the function of IL-33 on T cells, which may be one of the reasons for the T cell dysfunction in early HIV infection. Collectively, this data extends our understanding of the regulation of T cell function by the IL-33/ST2 axis during HIV-1 infection.

The first main finding of this study is that IL-33 levels were increased in early HIV-infected patients and that IL-33 enhanced the HIV-specific T cell function. IL-33 attracted attention because, aside from its traditional role as an “alarmin,” it provokes pleiotropic responses in different kinds of immune cells (8). Given the critical function of IL-33 in regulating T cell immune response in mice, it is important to know whether IL-33 has a role in regulating T cell function in HIV infection. Previous studies on the alterations of IL-33 in HIV-infected patients showed inconsistent conclusions (10, 13, 15). We found the expression of IL-33 levels was increased in early HIV infected patients compared to the healthy controls, which is in line with previous research (15). The elevation of IL-33 has been observed in a number of types of cancers (23–25) and other chronic viral infection, such as chronic hepatitis B infection (26). Our study was not consistent with Miyagaki T et al.'s and Mehraj V et al.' study (10, 13), which didn't find the elevated expression of IL-33 in HIV infection. All these studies used ELISA kit to detect the expression levels of IL-33, so the methodology may not be the cause of the different results. We postulated that the differences in clinical characteristics of patients may lead to the different findings in these studies. For example, our study enrolled treatment naïve HIV infected patients. While all the HIV infected patients enrolled in Miyagaki T et al.'s study were on ART and accompanied by skin diseases such as HIV related eosinophilic folliculitis or sexually transmitted diseases such as syphilis. In addition, previous study showed the upregulation of IL-33 produced by HIV-1 clade B infected cells in comparison to clade C, under identical *in vitro* infection levels (27). The clade specific responses may also lead to the different findings of the studies, because patients enrolled in the different studies were from different regions, and the predominant subtypes in these regions were various.

We then study whether IL-33 can influence the function of T cells in HIV infected patients. Studies performed in animal models have showed that IL-33 augments effector T cell responses in LCMV infection, GVHD, and murine acute myeloid leukemia (11, 28, 29). Because the concentration of IL-33 in the local tissue and microenvironment is higher than that in plasma, the concentrations of IL-33 added in the study (at ng/ml level) was higher than that in the plasma of controls and HIV patients (at pg/ml level) (30, 31). Our data revealed that elevated IL-33 can augment T cell function in HIV infection by intracellular cytokine staining and IFN-γ ELISPOT assay. We found that T cell responses to CEF peptides were also increased by IL-33 stimulation in HIV-infected patients, suggesting that the function of IL-33 has the capacity to increase both antigen-specific and antigen non-specific responses. To be noticed, we showed that IL-33 cannot achieve the strong improvement of T cell functions. Our data indicates that only IL-33 cannot promote robust T cell function, but may has the potential to be a molecular adjuvant to enhance T cell immune response. We did not find that IL-33 can induce higher expression of TNF-α or IL-2 in gag stimulated CD8+ T cells (data not shown). Villarreal et al. showed mice vaccinated with IL-33 have higher frequencies of LCMV antigen-specific CD8+ T cells producing triple-positive IFN-γ+TNF-α+IL-2+ cytokines in the spleens (32). While co-immunization of IL-33 with a DNA vaccine expressing the TB antigen induces higher percentages of CD8+ T cell producing total IFN-γ, TNF-α, but not IL-2 cytokine response (33). Our *in vitro* data suggested that IL-33 has the potential to increase the CD8+T cell function, but it cannot achieve the overall improvement of CD8+T cell functions in HIV infection. Because most of the current studies on the effect of IL-33 on poly-functional CD8+T cells were using mouse model, whether IL-33 can induce the polyfunctional CD8+T cells from humans in infectious diseases needs to be further investigated.

Although IL-33 can enhance T cell function, we observed that the increased IL-33 level in EHI correlates positively with viral load. We postulated that the function of IL-33 in EHI may be masked due to the complex immune regulation. The second main finding of this study is that increased sST2 level attenuates the regulation of T cells by IL-33. Under normal circumstances, IL-33 binds the transmembrane receptor ST2L to interact with IL-1RacP and then induces signaling through the MyD88 adaptor, activating signaling pathways of MAP kinases and NFκB transcription factors (34, 35). In the presence of inflammation, sST2 is produced by multiplicate cell types, including mast cells and T cells. A large number of sST2 are present in the serum of patients with various inflammatory diseases and relate to disease severity (36–38). Our results show that the levels of sST2 in the plasma of early HIV-infected patients were higher than that of normal controls, which was consistent with previous research (10, 13). Additionally, patients with advanced disease progression have a higher level of sST2 than those with relatively higher CD4+T cells and lower viral load. Previous study has shown that mucosal barrier tissues such as gastrointestinal tract store large amounts of IL-33 that may be released upon tissue injury (28). In HIV infection, gut damage occurs early after infection (39), which can cause the elevation of IL-33 and sST2. Most likely that gut tissue damage in early HIV infection are linked to the elevation of IL-33 and sST2 observed in our study. We found that adding ST2 antibody significantly decreased the secretion of IFN-γ in CD4+ and CD8+T cells stimulated by IL-33. As a decoy receptor, sST2 can neutralize the activity of IL-33 (8). We postulate that sST2 is not a direct contributor to the T cell dysfunction observed in HIV infected patients; it possibly neutralizes the T cell-promoting action of IL-33. Our data supports the suggestion that enhanced IL-33 levels facilitate IFN-γ production. However, excessive secretion of sST2 likely neutralizes the promoting effect of IL-33 during HIV, resulting in a lack of net effect. However, it cannot be ruled out that IL-33 can still exert a local and timely limited effect (40).

There are limitations in this study. First, we did not observe the dynamic changes of IL-33/ST2 throughout the whole phase of HIV infection; Second, local or tissue effect of IL-33/ST2 was not studied due to the difficulties in getting the samples. Based on these reasons, we still can't make a solid conclusion whether IL-33/ST2 is good or not at the time of acute infection, which is similar with the findings of IFN-α in early HIV infection. In this study, we found that IL-33 can promote the function of T cells, suggesting that IL-33 may act as an immunoadjuvant at enhancing T cell function in a vaccine setting in HIV infection. While the sST2 should not be overlooked during IL-33-related interventions for HIV infection because increased sST2 expression attenuates the function of IL-33.

In conclusion, we report that IL-33 has the potential to regulate the function of T cells, which play an important role in HIV disease progression. The upregulated sST2 in EHIs is associated with advanced disease progression, whereas the elevated level of sST2 in EHI weakens the activity of IL-33. The high level of sST2 in patients with advanced disease progression may contribute to the dysfunction of T cells and rapid disease progression. This data broadens the understanding of HIV pathogenesis and provides critical information for HIV intervention.

## Ethics Statement

The ethics approval was obtained from the First Hospital of China Medical University, and all the investigated participants were informed about the collection of blood samples, and provided written consent prior to enrolment in the study.

## Author Contributions

HS, Z-NZ conceived and designed the experiments. YL, C-BS, and Y-LC performed the experiments. XW analyzed the data. Y-JF, Y-JJ, and H-BD contributed reagents, materials, and analysis tools. HS and Z-NZ wrote the paper.

### Conflict of Interest Statement

The authors declare that the research was conducted in the absence of any commercial or financial relationships that could be construed as a potential conflict of interest.

## References

[1] Walker BD Yu XG. Unravelling the mechanisms of durable control of HIV-1. Nat Rev Immunol. (2013) 13:487–98. 10.1038/nri3478 23797064

[2] Streeck H Schweighardt B Jessen H Allgaier RL Wrin T Stawiski EW. Loss of HIV-1-specific T-cell responses associated with very rapid HIV-1 disease progression. AIDS (2007) 21:889–91. 10.1097/QAD.0b013e3280f77439 17415054

[3] Jansen CA Piriou E Bronke C Vingerhoed J Kostense S van Baarle D. Characterization of virus-specific CD8(+) effector T cells in the course of HIV-1 infection: longitudinal analyses in slow and rapid progressors. Clin Immunol. (2004) 113:299–309. 10.1016/j.clim.2004.08.002 15507395

[4] Stacey AR Norris PJ Qin L Haygreen EA Taylor E Heitman J. Induction of a striking systemic cytokine cascade prior to peak viremia in acute human immunodeficiency virus type 1 infection, in contrast to more modest and delayed responses in acute hepatitis B and C virus infections. J Virol. (2009) 83:3719–33. 10.1128/JVI.01844-08 19176632 PMC2663284

[5] Huang X Liu X Meyers K Liu L Su B Wang P. Cytokine cascade and networks among MSM HIV seroconverters: implications for early immunotherapy. Sci Rep. (2016) 6:36234. 10.1038/srep36234 27830756 PMC5103227

[6] Ramirez LA Arango TA Thompson E Naji M Tebas P Boyer JD. High IP-10 levels decrease T cell function in HIV-1-infected individuals on ART. J Leukocyte Biol. (2014) 96:1055–63. 10.1189/jlb.3A0414-232RR 25157027 PMC4226794

[7] Mendez-Lagares G Lu D Merriam D Baker CA Villinger F Van Rompay KKA. IL-21 Therapy controls immune activation and maintains antiviral CD8(+) T cell responses in acute simian immunodeficiency virus infection. AIDS Res Hum Retroviruses (2017) 33:S81–92. 10.1089/aid.2017.0160 29140110 PMC5684667

[8] Cayrol C Girard JP. Interleukin-33 (IL-33): a nuclear cytokine from the IL-1 family. Immunol Rev. (2018) 281:154–68. 10.1111/imr.12619 29247993

[9] Liew FY Pitman NI McInnes IB. Disease-associated functions of IL-33: the new kid in the IL-1 family. Nat Rev Immunol. (2010) 10:103–10. 10.1038/nri2692 20081870

[10] Mehraj V Ponte R Routy JP. The dynamic role of the IL-33/ST2 axis in chronic viral-infections: alarming and adjuvanting the immune response. EBioMedicine (2016) 9:37–44. 10.1016/j.ebiom.2016.06.047 27397514 PMC4972565

[11] Bonilla WV Frohlich A Senn K Kallert S Fernandez M Johnson S. The alarmin interleukin-33 drives protective antiviral CD8(+) T cell responses. Science (2012) 335:984–9. 10.1126/science.1215418 22323740

[12] Kakkar R Lee RT. The IL-33/ST2 pathway: therapeutic target and novel biomarker. Nat Rev Drug Discov. (2008) 7:827–40. 10.1038/nrd2660 18827826 PMC4277436

[13] Miyagaki T Sugaya M Yokobayashi H Kato T Ohmatsu H Fujita H. High levels of soluble ST2 and low levels of IL-33 in sera of patients with HIV infection. J Invest Dermatol. (2011) 131:794–6. 10.1038/jid.2010.366 21150924

[14] Mehraj V Jenabian MA Ponte R Lebouche B Costiniuk C Thomas R. The plasma levels of soluble ST2 as a marker of gut mucosal damage in early HIV infection. Aids (2016) 30:1617–27. 10.1097/QAD.0000000000001105 27045377 PMC4900419

[15] He B Zheng L Zhou H He Y Chen Z Xiao S. Dynamic observation of IL-33 and its receptors in HIV patients who received HAART. Cell Mol Biol. (2017) 63:73–7. 10.14715/cmb/2017.63.3.14 28466817

[16] Younas M Psomas C Mehraj V Cezar R Portales P Tuaillon E. Plasma level of soluble ST2 in chronically infected HIV-1 patients with suppressed viremia. Open AIDS J. (2017) 11:32–35. 10.2174/1874613601711010032 28553430 PMC5427803

[17] Secemsky EA Scherzer R Nitta E Wu AH Lange DC Deeks SG. Novel biomarkers of cardiac stress, cardiovascular dysfunction, and outcomes in HIV-infected individuals. JACC Heart Fail. (2015) 3:591–9. 10.1016/j.jchf.2015.03.007 26164679 PMC4529774

[18] Thiebaut R Hue S Le Marec F Lelievre JD Dupon M Foucat E. Serum suppression of tumorigenicity 2 level is an independent predictor of all-cause mortality in HIV-infected patients. AIDS (2017) 31:2355–65. 10.1097/QAD.0000000000001628 29068834

[19] Yang Q Li G Zhu Y Liu L Chen E Turnquist H. IL-33 synergizes with TCR and IL-12 signaling to promote the effector function of CD8+ T cells. Eur J Immunol. (2011) 41:3351–60. 10.1002/eji.201141629 21887788 PMC3332117

[20] Schiering C Krausgruber T Chomka A Frohlich A Adelmann K Wohlfert EA. The alarmin IL-33 promotes regulatory T-cell function in the intestine. Nature (2014) 513:564–8. 10.1038/nature13577 25043027 PMC4339042

[21] Hoffmann M Pantazis N Martin GE Hickling S Hurst J Meyerowitz J. Exhaustion of activated CD8 T cells predicts disease progression in primary HIV-1 infection. PLoS Pathog. (2016) 12:e1005661. 10.1371/journal.ppat.1005661 27415828 PMC4945085

[22] Du LX Wang YQ Hua GQ Mi WL. IL-33/ST2 Pathway as a rational therapeutic target for CNS diseases. Neuroscience (2018) 369:222–30. 10.1016/j.neuroscience.2017.11.028 29175156

[23] Sun P Ben Q Tu S Dong W Qi X Wu Y. Serum interleukin-33 levels in patients with gastric cancer. Dig Dis Sci. (2011) 56:3596–601. 10.1007/s10620-011-1760-5 21643739

[24] Liu J Shen JX Hu JL Huang WH Zhang GJ. Significance of interleukin-33 and its related cytokines in patients with breast cancers. Front Immunol. (2014) 5:141. 10.3389/fimmu.2014.00141 24778632 PMC3985005

[25] Lu B Yang M Wang Q. Interleukin-33 in tumorigenesis, tumor immune evasion, and cancer immunotherapy. J Mol Med. (2016) 94:535–43. 10.1007/s00109-016-1397-0 26922618

[26] Wang J Cai Y Ji H Feng J Ayana DA Niu J. Serum IL-33 levels are associated with liver damage in patients with chronic hepatitis B. J Interferon Cytokine Res. (2012) 32:248–53. 10.1089/jir.2011.0109 22304300 PMC3366095

[27] Yndart A Kaushik A Agudelo M Raymond A Atluri VS Saxena SK. Investigation of Neuropathogenesis in HIV-1 Clade B and C infection associated with IL-33 and ST2 regulation. ACS Chem Neurosci. (2015) 6:1600–12. 10.1021/acschemneuro.5b00156 26110635

[28] Reichenbach DK Schwarze V Matta BM Tkachev V Lieberknecht E Liu Q. The IL-33/ST2 axis augments effector T-cell responses during acute GVHD. Blood (2015) 125:3183–92. 10.1182/blood-2014-10-606830 25814531 PMC4432012

[29] Qin L Dominguez D Chen S Fan J Long A Zhang M. Exogenous IL-33 overcomes T cell tolerance in murine acute myeloid leukemia. Oncotarget (2016) 7:61069–80. 10.18632/oncotarget.11179 27517629 PMC5308636

[30] Bourgeois E Van LP Samson M Diem S Barra A Roga S. The pro-Th2 cytokine IL-33 directly interacts with invariant NKT and NK cells to induce IFN-gamma production. Eur J Immunol. (2009) 39:1046–55. 10.1002/eji.200838575 19266498

[31] Stojkovic S Kaun C Basilio J Rauscher S Hell L Krychtiuk KA. Tissue factor is induced by interleukin-33 in human endothelial cells: a new link between coagulation and inflammation. Sci Rep. (2016) 6:25171. 10.1038/srep25171 27142573 PMC4855148

[32] Villarreal DO Svoronos N Wise MC Shedlock DJ Morrow MP Conejo-Garcia JR. Molecular adjuvant IL-33 enhances the potency of a DNA vaccine in a lethal challenge model. Vaccine (2015) 33:4313–20. 10.1016/j.vaccine.2015.03.086 25887087 PMC4546882

[33] Villarreal DO Siefert RJ Weiner DB. Alarmin IL-33 elicits potent TB-specific cell-mediated responses. Hum Vaccin Immunother. (2015) 11:1954–60. 10.1080/21645515.2015.1026499 26091147 PMC4635936

[34] Liew FY Girard JP Turnquist HR. Interleukin-33 in health and disease. Nat Rev Immunol. (2016) 16:676–89. 10.1038/nri.2016.95 27640624

[35] Schmitz J Owyang A Oldham E Song Y Murphy E McClanahan TK. IL-33, an interleukin-1-like cytokine that signals via the IL-1 receptor-related protein ST2 and induces T helper type 2-associated cytokines. Immunity (2005) 23:479–90. 10.1016/j.immuni.2005.09.015 16286016

[36] Bandara G Beaven MA Olivera A Gilfillan AM Metcalfe DD. Activated mast cells synthesize and release soluble ST2-a decoy receptor for IL-33. Eur J Immunol. (2015) 45:3034–44. 10.1002/eji.201545501 26256265 PMC4813659

[37] Lecart S Lecointe N Subramaniam A Alkan S Ni D Chen R. Activated, but not resting human Th2 cells, in contrast to Th1 and T regulatory cells, produce soluble ST2 and express low levels of ST2L at the cell surface. Eur J Immunol. (2002) 32:2979–87. 10.1002/1521-4141(2002010)32:10<2979::AID-IMMU2979>3.0.CO;2-5 12355452

[38] Teufelberger AR Nordengrun M Braun H Maes T De Grove K Holtappels G. The IL-33/ST2 axis is crucial in type 2 airway responses induced by Staphylococcus aureus-derived serine protease-like protein D. J Allergy Clin Immunol. (2017) 141:549–59.e7. 10.1016/j.jaci.2017.05.004 28532656

[39] Ponte R Mehraj V Ghali P Couedel-Courteille A Cheynier R Routy JP. Reversing gut damage in HIV infection: using non-human primate models to instruct clinical research. EBioMedicine (2016) 4:40–9. 10.1016/j.ebiom.2016.01.028 26981570 PMC4776249

[40] Travers J Rochman M Miracle CE Habel JE Brusilovsky M Caldwell JM. Chromatin regulates IL-33 release and extracellular cytokine activity. Nat Commun. (2018) 9:3244. 10.1038/s41467-018-05485-x 30108214 PMC6092330

