# Corrigendum: Increased Expression of sST2 in Early HIV Infected Patients Attenuated the IL-33 Induced T Cell Responses

**DOI:** 10.3389/fimmu.2020.00088

**Published:** 2020-02-18

**Authors:** Xian Wu, Yao Li, Cheng-Bo Song, Ya-Li Chen, Ya-Jing Fu, Yong-Jun Jiang, Hai-Bo Ding, Hong Shang, Zi-Ning Zhang

**Affiliations:** ^1^NHC Key Laboratory of AIDS Immunology, Department of Laboratory Medicine, The First Affiliated Hospital of China Medical University, Shenyang, China; ^2^Department of Laboratory Medicine, The First Affiliated Hospital of Xiamen University, Xiamen, China; ^3^Clinical and Emergency Medical Laboratory Department, The First Hospital of Shanxi Medical University, Taiyuan, China; ^4^Collaborative Innovation Center for Diagnosis and Treatment of Infectious Diseases, Hangzhou, China; ^5^Key Laboratory of AIDS Immunology of Liaoning Province, The First Affiliated Hospital of China Medical University, Shenyang, China; ^6^Key Laboratory of AIDS Immunology, Chinese Academy of Medical Sciences, Shenyang, China

**Keywords:** IL-33, ST2, T cell response, IFN-γ, HIV infection

In the original article, there was a mistake in Figure 2C as published. The leftmost diagram at the bottom of Figure 2C was mistakenly duplicated from the third diagram at the bottom of Figure 2C during the figure preparation. The corrected Figure 2 appears below.

**Figure 2:**
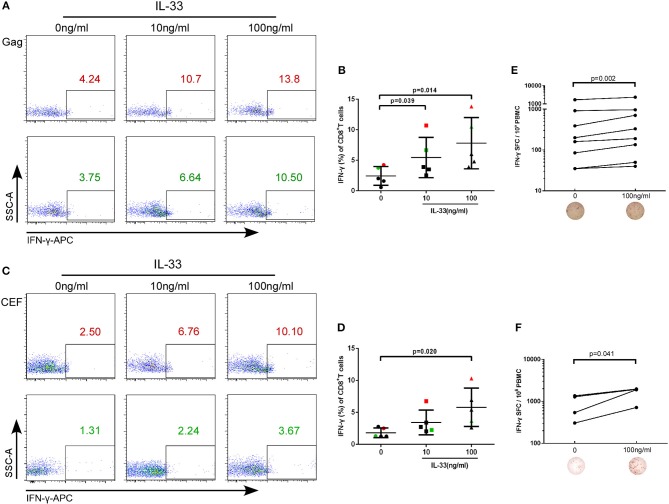
IL-33 increases the expression of IFN-γ by Gag and CEF stimulated CD8^+^ T cells. CD8^+^ T cells were isolated from HIV-1 individuals and treated with Gag peptide pools with rhIL-33 (10 ng/mL and 100 ng/mL) or without IL-33 (0 ng/mL). Intracellular IFN-γ expression was detected by flow cytometer and compared by paired *t*-test (0 ng/mL: 2.44 ± 1.53%; 10 ng/mL: 5.46 ± 3.30%; 100 ng/mL: 7.81 ± 4.20%). Representative flow cytometry dot plot **(A)** and summary data **(B)** were shown. CD8^+^ T cells were isolated from HIV-1 individuals and treated with CEF peptide pools with rhIL-33 (10 ng/mL and 100 ng/mL) or without IL-33 (0 ng/mL). Intracellular IFN-γ expression was detected by flow cytometer and compared by paired *t*-test (0 ng/mL: 1.81 ± 0.75%; 10 ng/mL: 3.44 ± 1.93%; 100 ng/mL: 5.80 ± 3.00%). Representative flow cytometry dot plot **(C)** and summary data **(D)** were shown. CD8^+^ T cells were stimulated with Gag peptide pools **(E)** or CEF peptides **(F)** and IFN-γ secretion was detected by ELISPOT assay. The numbers of spot forming cells (SFC) were log transformed and then compared by paired *t*-test. The number of SFC treated by 100 ng/mL IL-33 were compared with cells without IL-33 stimulation (0 ng/mL).

The authors apologize for this error and state that this does not change the scientific conclusions of the article in any way. The original article has been updated.

